# Plant Food Dyes with Antioxidant Properties and Allergies—Friend or Enemy?

**DOI:** 10.3390/antiox12071357

**Published:** 2023-06-28

**Authors:** Kinga Lis, Zbigniew Bartuzi

**Affiliations:** Department of Allergology, Clinical Immunology and Internal Medicine, Ludwik Rydygier Collegium Medicum in Bydgoszcz, Nicolaus Copernicus University in Toruń, ul. Ujejskiego 75, 85-168 Bydgoszcz, Poland

**Keywords:** allergy, anthocyanins, betanin, chlorophylls, carotenoids, curcumin

## Abstract

Color is an important food attribute which increases its attractiveness, thus influencing consumer preferences and acceptance of food products. The characteristic color of fresh, raw food is due to natural dyes present in natural food sources. Food loses its natural color during processing or storage. Loss of natural color (e.g., graying) often reduces the appeal of a product to consumers. To increase the aesthetic value of food, natural or synthetic dyes are added to it. Interestingly, the use of food coloring to enhance food attractiveness and appetizing appearance has been practiced since antiquity. Food coloring can also cause certain health effects, both negative and positive. Dyes added to food, both natural and synthetic, are primarily chemical substances that may not be neutral to the body. Some of these substances have strong antioxidant properties. Thanks to this activity, they can also perform important pro-health functions, including antiallergic ones. On the other hand, as foreign substances, they can also cause various adverse food reactions, including allergic reactions of varying severity and anaphylactic shock. This article discusses food dyes of plant origins with antioxidant properties (anthocyanins, betanins, chlorophylls, carotenoids, and curcumin) and their relationship with allergy, both as sensitizing agents and immunomodulatory agents with potential antiallergic properties.

## 1. Colorants as Food Additives

Color plays a significant role in the food production and processing sector, contributing to the sensory properties of food. Consumers often pay attention mainly to color when choosing food products. The color of food is related to its freshness, nutritional value, and safety. Food with an intense color is perceived as healthier [1].

Coloring food is supposed to increase its aesthetic value. Dyeing leads to the production of characteristic features of the product that enable its identification and are related to its use or intended use (e.g., candies, confectionery, desserts, soft drinks, flavored vodkas). Dyeing also restores the natural color of products that have lost their attractive color as a result of processing due to the degradation of natural dyes (e.g., graying of green peas during preservation). Dyes are also added to mask unfavorable discolorations or to reduce the loss of fragrance compounds and vitamins sensitive to light (e.g., the intense color of drinks in clear glass bottles prevents deeper penetration of sunlight and the breakdown of nutrients contained in it) [2,3,4].

The use of dyes as food additives is not a modern invention and dates back to ancient times. As early as 1500 B.C.E. the Romans and Egyptians colored wines, medicines, and various other everyday foods [5]. It is estimated that until around the mid-19th century, most food colorings came from natural sources such as peppers, blueberries, leaf chlorophyll, turmeric, indigo, cochineal, saffron, and various flowers [6,7]. In 1885, the first synthetic dye, fuchsine, was obtained, which began the era of synthetic dyes [8]. It is worth noting that dyes from natural sources were very expensive and difficult to obtain. It is believed that the development of fuchsin synthesis technology has opened the way to obtaining various dyes for both food and textile applications on a large scale at relatively low costs. Officially, the beginning of the era of the industrial use of synthetic dyes is considered to be 1856, when William Henry Perkin, looking for a simple way to synthesize quinine, accidentally obtained an intense purple dye—mauveine—and patented his invention [9]. Mauveine is considered to be the first organic synthetic dye to be used on an industrial scale. After this discovery, the use of expensive and unstable natural dyes was discontinued, replacing them with synthetic dyes. Due to chemical stability, low production costs, and a larger range of shades, synthetic dyes were willingly used. Initially, they were mainly used for dyeing textiles [10]. It was noticed that synthetic dyes have very strong coloring properties, so obtaining intense colors requires the addition of small amounts of dye. Due to this property, they were also used for coloring food products. Unfortunately, the first dyes of this type were aniline derivatives, which is a toxic compound, and its consumption can have dangerous side effects [5,7].

Suspicions as to the harmful effects on humans of dyes added to food and the first legal regulations prohibiting food adulteration with dyes date back to 14th century France. In turn, the English chemist Friedrich Accum [11] was the first to draw public attention to the problem of poisonous dyes added to food (e.g., lime, copper, or lead), which were supposed to suggest its more luxurious origin. In 1820, Accum published a book that focused on food and culinary poisons. The book contained examples of food products contemporary to the author, in which poisonous dyes were used to mask the true nature of the product and make it more exclusive [11]. Currently, food colorings, both natural and synthetic, are thoroughly tested for safety. Their use is governed by the relevant laws in force in a particular country. In Europe, the rules for the use of food additives, including dyes, are regulated by the Regulation of the European Parliament 1333/2008 [12] and the food safety authority is the European Food Safety Authority (EFSA) [13]. 

## 2. The Aim of the Manuscript and Methods of Its Implementation

The aim of the manuscript was to discuss plant-derived food colors with antioxidant properties (anthocyanins, betanin, chlorophylls, carotenoids, and curcumin) and their relationship to allergy, both as sensitizing and immunomodulatory agents with potential antiallergic properties.

In order to implement the assumptions, the chemical properties and origin of individual dyes as well as their generally recognized, general health-promoting properties were described. Then, based on the PubMed database, the available literature data on reported clinical cases of allergy were analyzed, in which the relationship between the observed clinical symptoms and a specific food color additive was documented or proved to be very probable. On the other hand, data (also based on the PubMed database) were also analyzed examining the antiallergic effect of food dyes with antioxidant properties and their importance in the prevention and treatment of allergies.

The following terms were used in the data search strategy: food dyes and allergy, food dyes and hypersensitivity, anthocyanins and allergy, anthocyanins and hypersensitivity, betanin and allergy, betanin and hypersensitivity, chlorophyll and allergy, chlorophyll and hypersensitivity, chlorophyllins and allergy, chlorophyllins and hypersensitivity, carotenoids and allergy, carotenoids and hypersensitivity, lycopene and allergy, lycopene and hypersensitivity, curcumin and allergy, and curcumin and hypersensitivity. Additional entries were also used, which resulted from previously found data, e.g., grapes and allergy, grapes and hypersensitivity, beetroot and allergy, beetroot and hypersensitivity, etc.

## 3. Antioxidant Dyes as Functional Food Additives and Their Relationship with Allergy

In the past, attention was drawn to the fact that dyes added to food were primarily intended to improve the appearance of food, and not to harm consumers. Recently, the approach to food additives has changed. It is important not only that food additives fulfill their technological function and are safe for consumers, but the additional functionality of the added substances, which has become an added value to their main purpose, is also very important. According to current trends, food additives, in addition to improving the organoleptic properties of food, should also increase the nutritional value of food. Food colorings have, for example, antioxidant properties [14]. Antioxidants are perceived as substances with significant health-promoting properties. Food supplementation with antioxidants, including antioxidant dyes, may increase the nutritional value of foods [15,16]. In unprocessed food, the most valuable sources of antioxidants are blue, red, and yellow fruits and vegetables, rich in anthocyanins and carotenes. A less important source of antioxidants are the green parts of plants, which are rich in chlorophyll [14]. 

Dyes of natural origin, including antioxidants, the source of which is mainly plant material, usually do not raise health concerns. An analysis by Lucas et al. [17] showed that adverse reactions, including allergies, to natural food dyes are rather rare. Interestingly, there are various, contradictory hypotheses as to the possible relationship of antioxidant dyes added to food with allergic diseases, according to which these additives can both sensitize and prevent sensitization. In addition, it is also considered that the increase in the incidence of allergic diseases may be a consequence of both low and, on the contrary, high consumption of antioxidants. Since antioxidants are biologically active substances, it cannot be ruled out that all these hypotheses are possible [18,19,20]. 

### 3.1. Anthocyanins

Anthocyanins are a group of glycosidic dyes belonging to flavonoids, i.e., polyphenolic organic compounds. Polyphenols are a large group of dyes included in the so-called natural non-nutritive substances of plant origin. They are soluble in water. Currently, several hundred natural anthocyanin dyes and over a hundred synthetically obtained ones are known. Anthocyanins are found in various parts of plants; in flowers, fruit pulp, skin, leaves, stems, roots, and wood. Plant tissues usually contain a few to a dozen different anthocyanins. These dyes are characterized by a very diverse color from orange through various shades of red and violet to blue. Anthocyanins are chemically unstable. In the aqueous environment, depending on the pH and the presence of metal ions, anthocyanins change color due to a change in the structure of their molecules. In an acidic environment (pH < 3) anthocyanins are red, in a neutral environment (pH 7) they are violet, and in an alkaline environment (pH > 11) they are blue. Anthocyanins have strong antioxidant, anti-inflammatory, and anticancer properties. For this reason, anthocyanins are used as an active ingredient in nutraceuticals [21,22,23]. 

Natural anthocyanin dyes are most often obtained from grape pomace, blackcurrant, blueberries, elderberry, chokeberry, and cranberry. Recently, an extremely popular source of intensely blue anthocyanins is *Clitoria ternatea* (*Clitoria ternatea* L.)—a species of tropical plant from the Fabaceae family. Anthocyanins as food colors are mainly used for the production of desserts and drinks. Blue food is considered extravagant and playful. Most often, extracts that are a mixture of anthocyanins are used, not single compounds. It is a simpler and cheaper solution. An interesting fact is that anthocyanin dyes are also used as indicators to assess the quality of colored food. The assessment of the anthocyanin profile is used to assess the quality of fruit jams and to check the quality of wine. The aging of these products causes a characteristic color change. It is associated with changing pH which is associated with the aging process of food [24,25,26]. 

Anthocyanins and their metabolites (mainly monoglucuronides) are excreted by the kidneys. These dyes are detected in the urine up to 24 h after ingestion [27]. In the available literature, there are few reports of allergic reactions associated with the consumption of anthocyanins, either naturally occurring in food or added to food for coloring. Gallo et al. [28] observed that the purple anthocyanin from eggplant skin, nasunin, can cause local hypersensitivity reactions. In patch tests conducted by these researchers on healthy volunteers with nasunin-containing eggplant peel extract, 12% of subjects experienced moderate skin irritation and 3% experienced an allergic reaction. Positive reactions were observed at a concentration of the dye above 5%, while the concentration of this dye in food or cosmetics usually does not exceed 1%. It seems that nasunin is a safe dye for food, medicine, cosmetics, and textiles [28].

Anthocyanins are naturally occurring pigments in various fruits and vegetables. Therefore, it is possible that they may be the cause of allergic reactions associated with the consumption of naturally containing plant foods. Case reports of allergic reactions of various clinical manifestations after consumption of grapes, especially purple or red wine, often in the presence of various cofactors, are available. Sebastia et al. [29] reviewed 30 publications (1999–2010) on grape allergy. They reported both cases of oral allergy syndrome (OAS) and systemic anaphylactic reactions, usually in the presence of a cofactor (exercise or alcohol). It is worth noting that, in the analyzed cases of allergy to grapes or red wine, anthocyanins were never considered as the cause of hypersensitivity. Grapes contain a wide variety of well-identified proteins with known allergenic potential, such as lipid transfer proteins (nsLTP; Vit v 1), thaumatin-like proteins (TLPs), endochitinase 4A (the main grape allergen mainly responsible for allergic reactions to red wine), and endochitinase B [30]. Sensitization to any of these proteins may cause a strong systemic anaphylactic reaction after consumption of grapes or their products (e.g., red wine), regardless of the grapes’ natural anthocyanins. The anthocyanins naturally present in grapes are not considered allergens.

An interesting edible source of anthocyanins are the flowers of *Clitoria ternatea* (butterfly pea). They are mainly available in the form of dried leaves, from which intense blue infusions are obtained, known as blue tea (butterfly pea tea) with a characteristic herbal, earthy-woody taste. Clitoria flower infusion is also used as a natural blue colorant for drinks and desserts. Clitoria flowers can also be used to produce infusions in colors other than blue, by modifying the pH of the extract (e.g., lemon juice added to blue tea changes its color to purple) [31,32]. 

*Clitoria ternatea* is not only the source of the naturally rare blue dye, but also an important remedy in traditional Ayurvedic medicine in Asia [33]. In traditional medicine, the anti-inflammatory and antidiabetic effects of *Clitoria* root, seed, leaf, and flower extracts are mainly used. Both the color and the anti-inflammatory properties of *C. ternatea* petals are due to the high content of anthocyanins, mainly teratins-polyacylated delphinidin derivatives [32,34]. The healing properties of *Clitori* aextracts used in traditional medicine have been confirmed by the results of current research. Nair et al. [35] identified twelve phenolic metabolites (nine ternatin anthocyanins and three glycosylated quercetins) in *Clitoria ternatea* blue flower extracts. All *Clitoria ternatea* polyphenols exhibited anti-inflammatory properties in macrophage-mediated inflammation caused by lipopolysaccharides (LPS). It has been shown that *Clitoria ternatea* flower extracts strongly inhibit the activity of cyclooxygenase-2 (COX-2), reduce the release of free oxygen radicals, and reduce the production of nitric oxide by reducing the expression of nitric oxide synthase (iNOS). The available research results [32,35,36,37,38] of extracts from other plants rich in ternatins indicate that they are a very good source of natural antioxidants, thanks to which they have a strong anti-inflammatory and antianaphylactic effect [32,35,36,37,38].

The available data do not indicate that intense blue *Clitoria ternatea* flower extracts cause hypersensitivity reactions, regardless of the route of exposure. There are no reports of allergies after consuming infusions of these flowers or any food colored with them. On the contrary, it seems that extracts from various parts of clitoria (flowers, roots, or seeds) may have antihistamine and antiasthmatic effects [32,39]. Singh et al. [40] observed in an animal model that an alcoholic extract of *Clitoria ternatea* flowers reduces dyspnoea and bronchospasm provoked by asthma exacerbation and reduces the concentration of proinflammatory cytokines, i.e., interleukin 1 beta (IL-1 beta) and interleukin 6 (IL-6) in the bronchi and alveoli. Therefore, it seems that standardized *Clitoria* flower extracts may be a potential supportive therapy in the treatment of allergic asthma.

In summary, anthocyanins present in food, both naturally and added to foods for color, appear to have antiallergenic rather than proallergenic properties. The GA²LEN study showed that regular consumption of foods rich in anthocyanins, proanthocyanidins, and flavonoids not only does not involve a significant risk of allergic reactions, but also has a positive effect on the respiratory system and improves respiratory parameters measured by spirometry [41].

### 3.2. Betanin

Betanin, also called beetroot red, is an organic chemical compound (glycoside) from the group of betalains. It is a natural food coloring with a dark red to purple color. Betanin is obtained from beets. Betanin dissolves in water and is sensitive to light and high temperature. These properties significantly limit the possibilities of using betanin for food coloring. Betanin is usually used in the dairy industry, mainly to color ice cream and yogurt. It is considered a completely harmless substance that can be consumed without restrictions. Under physiological conditions, it is completely excreted from the body in the urine [42,43]. The only case of hypersensitivity to betanin (beetroot red) was reported by Zenaidi et al. [44] based on prospective studies. Moreover, according to studies by Li et al. [45], betanin from food may have a protective effect against the development of allergic asthma. Li et al. [45] examined the effect of betanin on experimentally induced ovalbumin (OVA) allergic asthma in BALB/c mice. It was observed that daily dietary supplementation with batanin significantly reduced the laboratory and clinical markers of allergic asthma and had a beneficial effect on the intestinal microbiota profile in mice. Also, Wang et al. [46] confirmed the immunomodulatory and anti-inflammatory properties of betanin. It seems, therefore, that a diet rich in betanin prevents the development of allergic asthma and may be an effective support in the treatment of this disease.

### 3.3. Chlorophylls

Chlorophylls are natural lipid pigments present, e.g., in parts of plants exposed to light, algae, and photosynthetic bacteria (e.g., cyanobacteria). These are the basic pigments that enable the conversion of light energy into high-energy compounds in the process of photosynthesis. Chlorophylls are typical metalloporphyrin compounds (similar to hemoglobin and myoglobin). They are responsible for the characteristic green color of the organisms in which they occur. There are many types of chlorophylls (denoted by the letters a through g). Chlorophyll a (blue-green) and chlorophyll b (yellow-green) are the most abundant in nature. Under natural conditions, chlorophylls a and b occur together in a ratio of about 3:1 in all photosynthetic plants [47,48,49].

Chlorophylls for industrial applications are obtained as a result of solvent extraction of natural varieties of edible plant materials, including grasses, alfalfa, and nettle. The main color components are pheophytins and magnesium chlorophylls. Phaeophytins are formed as a result of partial or complete removal of naturally occurring coordination magnesium from chlorophylls in the process of removing post-extraction solvents. It should be remembered that the chlorophyll obtained as a result of extraction also contains other dyes, such as carotenoids, oils, fats, and waxes derived from natural raw material. Due to the instability of coordinated magnesium in the chlorophyll molecule and the associated color change from olive green to dark green, the use of chlorophyll as a food coloring is very limited. Chlorophyllins are derivatives of chlorophylls. Chlorophyllins are obtained via alkaline hydrolysis and cleavage of phytol from the chlorophyll molecule. They are more stable than chlorophylls and less sensitive to changes in the pH of the environment. This makes them better dyes for industrial applications than chlorophylls [47,48,50,51]. In addition to chlorophylls and chlorophyllins, their copper complexes are also used as food dyes [50,51,52,53].

Chlorophylls dissolve in fats and chlorophyllins dissolve in water [51]. Chlorophylls and chlorophyllins and their copper complexes are used as color additives in pasta, flavored vegetable oils, ice cream, fruit jellies, beverages, candies, canned peas, and pharmaceuticals and cosmetics. Chlorophyll dyes are considered the least durable plant dyes. They retain their characteristic green color only in living, undamaged tissues. The rate and nature of changes occurring during the storage and processing of raw materials rich in chlorophylls depends on environmental conditions, including mainly temperature, acidity, oxygen availability, the presence of metals, and specific enzymes such as chlorophyllase and lipoxygenase. Chlorophyllins are less sensitive to environmental conditions and have more intense colors than chlorophyll. [47,49,51,52,53,54]. 

Chlorophyll and its derivatives and metabolites are believed to have antioxidant, anti-inflammatory, and antiviral properties. Eating foods with a high content of these dyes may have a beneficial effect on health [47,51,52,53,54,55,56,57,58,59,60]. For example, the phyllobilins (e.g., phylloucobilin, dioxobilin-type phylloucobilin, and phylloxanthobilin) are natural products of chlorophyll degradation. These linear chlorophyll metabolites are strong antioxidants with interesting physiological properties, including immunomodulatory ones [61]. Although the exact mechanisms of the immunomodulatory effects are yet unknown, Karg et al. [55] experimentally demonstrated the dose-dependent ability of phytobilins to inhibit the catabolism of tryptophan to kynurenine, suggesting a suppressive effect on cellular immune activation pathways. It is also possible that chlorophylls in combination with other substances, including chemotherapeutics, may show a synergistic effect and enhance the therapeutic effect of these substances. The results of Lauritano et al.’s [56] experiments also showed that the products of the metabolic degradation of chlorophyll can inhibit the secretion of tumor necrosis factor alpha (TNF-α) using lipopolysaccharide (LPS)-stimulated macrophages. Also, Lin et al. [57] observed the anti-inflammatory effect of chlorophyll and its derivatives by reducing the synthesis of proinflammatory factors and reducing the adhesion of inflammatory cells to the aortic endothelium in a mechanism dependent on the secretion of TNF-α by monocytes. Wang et al. [58], based on the results of their own research, postulate that chlorophylls can be a natural means of increasing the effectiveness of chemotherapeutic agents in the case of multi-drug-resistant pathogens. It is also likely that chlorophylls play a key role in the anticancer properties of natural plant extracts. Uğuz et al. [59] showed that the removal of chlorophylls from green tea and olive leaf extracts significantly reduced the toxic effect of these solutions on cancer cells. In the light of the available data, it seems that chlorophyll and its derivatives have a huge pro-health potential, both individually and by enhancing the effect of other active food substances.

Despite the expected benefits of coloring food with chlorophyll and its derivatives, it is worth asking whether this supplementation is not associated with the risk of allergic reactions in consumers. The small number of available reports of possible allergic reactions associated with the consumption of chlorophyll suggests that this dye does not pose a significant allergic risk. In fact, only Böhm et al. [60] in 2001 reported a case of a 28-year-old woman with allergy symptoms (recurrent angioedema, rhinoconjunctivitis, asthma-like symptoms), which they linked to the consumption of green-colored jelly beans with copper complexes of chlorophyll and chlorophyllins. The patient underwent two challenge tests: one open (with green jelly beans) and one blinded, placebo-controlled (with copper chlorophyllin), during which an anaphylactoid reaction in the form of facial angioedema and a runny nose was observed. Symptoms appeared 10 min after ingestion of five green jelly beans or 1 mg of copper chlorophyllin. As recommended, the patient eliminated copper chlorophyllin-stained foods from her diet and no new episodes of angioedema were observed during the three-year follow-up.

The problem of allergy to chlorophyll is not only the hypersensitivity to food color additives, but also the possible allergenic properties of chlorophyll naturally occurring in plants. Valbuena et al. [62] reported a clinical case of a 52-year-old housewife with a history of allergic rhinoconjunctivitis to grass pollen, who experienced two episodes of shortness of breath with wheezing and coughing within 8–10 h after preparing raw chard. She had never experienced symptoms after inhaling chard fumes or after touching or ingesting chard before. Both raw chard and chlorophyll provocation results were positive in the patient. According to the clinical history, specific IgE for birch profilin (Bet v 2) was measured, but the result of this test was negative. Finally, Valbuena et al. [62] diagnosed the patient as cross-allergic to a plant allergen other than profilins, but did not clearly identify the causative allergen.

When analyzing the case described above [62], it should be taken into account that natural sources of chlorophyll may contain proteins other than profilins, which may also cause allergies. Another case of chard allergy was reported by Jara-Gutiérrez et al. [63]. These authors described a severe allergic reaction (cough, conjunctivitis, and angioedema) that developed in a 54-year-old woman minutes after contact with raw chard. The patient underwent skin prick tests (SPT) with a series of common inhalant allergens, lipid transfer proteins (LTP), and profilins, and native prick tests with raw chard, spinach, sugar beet, lettuce, and onion. The results were positive for pollen from grasses, olives, cypresses, plantains, Swiss chard, and profilins. Specific IgE was measured for beetroot (0.84 kU/L) and spinach (<0.1 kU/L). The test results suggested an allergy to profilin, which was not consistent with the patient’s clinical symptoms (severe reaction after contact with chard and no symptoms of inhalant allergy). As a result of subsequent laboratory tests, including the inhibition reaction on blots from raw and cooked beetroot extracts, a diagnosis of sensitization to chlorophyll a/b binding protein in chloroplasts with a molecular weight of 28 kDa was given.

Chlorophyll-binding proteins in chloroplasts are recognized allergens. Although to date only one allergen belonging to this group of proteins (Api g 3, celery) has been well-characterized and registered, it is very likely that proteins binding chlorophyll from other plants may also have significant allergenic properties.

Chlorophylls and their metabolites present in food can theoretically also have an antiallergic effect due to the antioxidant properties of these compounds [64]. Fujiwara et al. [65] noted that chlorophyll c2 extracted from Sargassum horneri reduced allergic symptoms in an animal model of allergic rhinitis. These researchers also evaluated the effectiveness of chlorophyll c2 in the treatment of patients with seasonal allergic rhinitis. This was a single-center, randomized, double-blind, placebo-controlled study. Sixty-six patients (20–43 years) with a minimum of 2 years of clinical history of allergic rhinitis were randomized to receive a single daily dose (0.7 mg) of chlorophyll c2 or placebo for 12 weeks. Throughout the procedure (weeks 4, 8, and 12), patients’ need for antiallergy medications (H1 antihistamines and nasal corticosteroids) and disease-specific quality of life (Japan Rhinitis Quality of Life Questionnaire; JRQLQ) were monitored. The results were compared to the state from before the start of the procedure. Based on the results, Fujiwara et al. [65] concluded that chlorophyll c2 appears to be an attractive alternative treatment for allergic rhinitis.

### 3.4. Carotenoids

Carotenoids are a wide group of yellow-orange plant pigments with a wide range of applications in food. Carotenoids include carotenes (including β-carotene, β-8′-apocarotenal, β-apo-8′-carotenic acid ethyl ester, annatto, capsanthin, capsorubin, capsaicin, and lycopene) and xanthophylls (lutein, canthaxanthin). More than 700 carotenoids have been identified, yet only 40 are included in the human diet, with β-carotene, α-carotene, lycopene, β-cryptoxanthin, lutein, and zeaxanthin the most common. Carotenoids are mainly plant pigments. Fruits and vegetables are the main source of carotenoids in the human diet. α- and β-carotene (yellow to red) are the main pigments in carrots, oranges, peppers, and leafy vegetables. Lycopene (bright red) is the main carotenoid in tomatoes, but it also gives color to the flesh of papaya, guava, and watermelon. β-cryptoxanthin (yellow to orange) is found in citrus fruits, melon, potato, guava, and apple. Lutein and zeaxanthin isomers (yellow) color corn and marigold flowers as well as pumpkin and pepper flesh, broccoli flower, and pulses. Xanthophylls, violaxanthin (yellow), capsanthin, and capsorubin (orange to red) are the main pigments in peppers. Some carotenoids are found only in algae and seafood, for example, astaxanthin (pink-red) colors krill, shrimp, and flamingo feathers, and fucoxanthin (brown) is the main color of brown algae [66,67,68]. 

Carotenoids are polyene compounds built of isoprene units. Carotenoids are fat-soluble and xanthophylls are also alcohol-soluble. The most common carotenes in the plant world are carotenes (α-, β-, γ-) and lycopene. Of the xanthophylls, the most common are lutein, zeaxanthin, canthaxanthin, and capsanthin. Carotenes and xanthophylls are used in the food industry as food color additives. They are usually added to yoghurts, milk drinks, homogenized cheeses, milk desserts, condensed milk, cream, processed cheeses, and coffee and tea whiteners. They are also used as an additive to feed for chickens to obtain egg yolks with an intense yellow-orange color [69,70]. 

All carotenoids are strong antioxidants and have anti-inflammatory, anticancer, and antiaging properties documented by relevant studies [64,71,72,73]. Allergy to carotenoids, if any, appears to be extremely rare. Few reports of allergic reactions involving carotenoids, mainly annatto pigment, have been reported.

Annatto (bixin and norbixin) is an orange-yellow food coloring from the seeds of the Bixa orellana tree (properly Arnota, the tree of Orleans). It is commonly used in cheeses, snacks, smoked fish, beverages, bakery products, and cereals. The most commonly reported adverse reactions associated with annatto dye were anaphylaxis with urticaria and angioedema [74,75,76,77]. Cis-bixin extracted from annatto is a contact allergen, which was confirmed by experiments on an animal model [78]. It is also suspected that this dye may cause symptoms of irritable bowel syndrome (IBS) [79,80].

It should also be noted that annatto dye, as a natural seed extract, may be contaminated with various proteins naturally found in these seeds. These proteins may be the standalone cause of IgE-mediated hypersensitivity, with or without dye. Plant seeds are a rich source of storage proteins, which are strong and stable allergens that can cause severe allergic reactions in people who are allergic to them. Ramsay et al. [81] described an anaphylactic reaction to blackberry seeds. This reaction was confirmed by the positive results of native tests, carried out with a suspension of powdered blackberry seeds.

An interesting carotenoid pigment is lycopene. It is estimated that only about 50–65% of the lycopene consumed comes directly from fruits and vegetables. The source of lycopene are also colored food products, mainly flavored milk, confectionery, and soft drinks. [82,83,84]. Lycopene, like other carotenoids, is known for its antioxidant properties [71,83,84,85]. 

To date, no clinical case of an allergic reaction to lycopene has been reported. It is theoretically possible to contaminate lycopene extracts from natural sources with other plant source proteins. These proteins may have allergen properties and may cause hypersensitivity reactions, independent of lycopene. For example, tomatoes are a rich source of lycopene, but tomato is also a source of other proteins with potential allergenic properties, including strong allergens such as non-specific lipid transfer proteins (nsLTP) [86]. Currently, however, lycopene used to color food is most often a synthetic dyeidentical to the natural dye. Therefore, lycopene added industrially to food as an additional dye does not contain other ingredients that may cause allergic reactions [82,87].

Moreover, according to research by Hossin et al. [88], daily supplementation with lycopene-rich red tomato peel extract may reduce allergy symptoms. According to them, the lycopene present in this extract probably inhibits the release of histamine, although the mechanism of this phenomenon has not been explained. Also, Polat et al. [89] showed that lycopene was effective in the treatment of experimentally induced allergic rhinitis in rats, and this effect was stronger with increasing lycopene doses. In turn, Ushiroda et al. [90] showed that lycopene ingestion induces regulatory colonic T cells in mice and suppresses food allergy symptoms.

The results of the presented studies seem to indicate that lycopene may be a useful food additive in the prevention of allergies. It seems that supplementation with other carotenoids may also have similar beneficial antiallergic effects [74]. In 1997, Schmutzler et al. [91] showed that beta-carotene inhibits the secretion of histamine via human mast cells and monocytes.

### 3.5. Curcumin

Curcumin (diferuylmethane, diferuloylmethane) is one of the polyphenolic compounds present in turmeric root (*Curcuma longa*). Turmeric polyphenols make up about 5% of the weight of the root and are called curcuminoids. Curcuminoids, apart from curcumin, also include cyclocurcumin, demethoxycurcumin, and bisdemethoxycurcumin. All curcuminoids are active antioxidants, and curcumin is considered the most active of them [92,93,94]. Curcumin is also used as a yellow-orange food coloring. The curcumin molecule was first isolated from turmeric in 1815, and in 1910 its spatial structure was determined [95,96].

Turmeric (*Curcuma longa*) is a herbaceous plant from the ginger family. It is grown mainly in the south and south-west regions of tropical Asia. Turmeric powder is a well-known spice in Asian cuisine. It can be used alone or as a component of spice mixtures, e.g., curry [94]. 

Since the time of Ayurveda (1900 BC), turmeric has been credited with numerous therapeutic effects for a wide variety of diseases and conditions, including skin, respiratory and digestive disorders, pains, wounds, sprains, and liver disorders [97,98]. This plant is an herb commonly used in traditional medicine in India and China to treat conditions such as dermatological conditions, infections, stress, and depression [94]. Curcumin has been shown to have antioxidant, anti-inflammatory, antiviral, antibacterial, antifungal, and anticancer effects, and therefore has potential applications in the treatment of various diseases, diabetes, allergies, arthritis, Alzheimer’s, and other chronic diseases [94,97]. The anti-inflammatory mechanism of curcumin is not completely understood, and isprobably involved in the regulation of various transcription factors, growth factors, inflammatory cytokines, protein kinases, and other enzymes relevant to inflammation [94,97,98,99,100,101]. 

Based on the available information, curcumin does not appear to be the cause of allergic reactions. Currently, no cases of allergy have been described in which an allergy to this dye has been unequivocally confirmed. Irani et al. [102] in a letter to the editor described an interesting case of Stevens–Johnson syndrome (SJS) in a 50-year-old woman, which probably developed after repeated intake of curcumin. Laboratory studies showed normal hemoglobin, white blood cell count, and platelets. Biochemistry evaluation, C-reactive protein, and chest x-ray were normal. Serum protein electrophoresis showed hypergammaglobulinemia. Serology of cytomegalovirus and Herpes simplex virus showed high specific IgG and low IgM. IgM antimycoplasma pneumonia was low threshold with a negative IgG. Skin biopsy confirmed the presence of SJS. The woman recently started taking 2 teaspoons (5 g) of curcumin daily for health reasons. Other possible causes of SJS were excluded in the patient. She had no history of allergy, drug consumption, fever, or upper respiratory infection. Regular curcumin supplementation was the only change that occurred in the patient’s life. For this reason, curcumin was considered to be the cause of SJS in the woman. The patient was treated with antihistamines and intravenous methylprednisolone starting at 4 mg/kg per day for 5 days and then tapered very slowly as the eruption resolved. She was discharged after clinical remission on oral prednisone tapered and antihistamines. According to Irani et al. [102] the proapoptotic and antiproliferative properties of curcumin [103] may hypothetically facilitate or even cause the onset of SJS, a mast-cell-independent allergic reaction.

This hypothesis formulated by Irani et al. [102] seems to be contradicted by the results of the experiment reported by Ceylan et al. [104]. Ceylan et al. [104] in their experiment examined the effect of curcumin on the viability and proliferation of nasal epithelial cells. The procedure involved adding 2.5 µM curcumin to a primary culture of physiologically normal nasal epithelial cells. After 24 h of culture with the addition of curcumin, the viability and proliferative capacity of these cells were assessed. Both cell viability and proliferative capacity were shown to be unchanged. In addition, no damage to primary nasal epithelial cells was observed after topical application of curcumin. The beneficial effect of curcumin on the nasal mucosa has been studied in animal models [105,106,107,108]. The results of these experiments suggest that topical administration of curcumin (on the nasal mucosa) may be an interesting alternative in the treatment of allergic rhinitis or a therapy adjunct to the pharmacological treatment of this disease, due to its anti-inflammatory, immunomodulatory, and inhibitory effects on mast cell histamine secretion [105,106,107,108].

It seems that the antiallergic properties of curcumin may be related to its effect on the biology of cells involved in hypersensitivity reactions. The immunomodulatory effect of curcumin is probably mainly due to its interaction with a vast collection of immune cells, such as mast cells, eosinophils, epithelial cells, basophils, neutrophils, and lymphocytes [107,108,109,110]. According to the observations of Kong et al. [111], curcumin inhibits IgE-mediated mast cell degranulation, thereby limiting histamine secretion. It also reduces the expression of FcεRI receptors on the surface of basophils in vitro. It seems, therefore, that curcumin not only does not cause allergic reactions, but even has antiallergic properties. According to Chen et al. [112], curcumin and its derivatives can be used to support the treatment of asthma as part of the diet. Shin et al. [113] and Kinney et al. [114], in a mouse model of food allergy, observed that curcumin significantly alleviated the symptoms of the disease in animals. They suggest that curcumin as an antiallergic agent has an immune-regulating effect by maintaining the Th1/Th2 immune balance and lowering IgE levels. They suggest that the administration of curcumin may be useful in alleviating allergic disorders such as food allergy, atopic dermatitis, and asthma. According to Lin et al. [115], curcumin may have antiallergic and even antianaphylactic effects, as they showed that it inhibits cyclooxygenase-2 (COX-2) dependent on prostaglandin D 2 (PGD 2) and 5-lipoxygenase (5-LO) leukotriene C 4 (LTC 4) in a dose-dependent manner. In addition, curcumin inhibits intracellular Ca^2+^ influx through activation of phospholipase Cγ1 (PLCγ1) and phosphorylation of mitogen-activated protein kinases (MAPKs) and the nuclear factor κB (NF-κB) pathway. Zeng et al. [116] demonstrated in a mouse model that curcumin inhibits the release of β-hexosaminidase, interleukin-4, and tumor necrosis factor-α, which inhibits the degranulation of allergen-stimulated mast cells and the release of allergic reaction mediators (including histamine) from these cells viaIgE-mediated hypersensitivity reaction. 

In conclusion, the analysis of the available data suggests that curcumin not only does not cause allergic reactions, but on the contrary, it may be an attractive substance of natural origin, the immunomodulatory effect of which can be used, to support the treatment of asthma, food allergies, and anaphylaxis.

## 4. Pro- and Antiallergic Properties and Acceptable Daily Intake (ADI) of Selected Antioxidant Food Colors in Scientific Opinions of EFSA Expert Panels

The European Food Safety Authority, EFSA, is the European Union’s agency for providing independent scientific advice on existing and emerging risks in the food chain. This institution was established in 2002; its headquarters are in Parma [13].

The main area of the EFSA’s activities is the collection, evaluation, and integration of scientific evidence on food safety and risk assessment. The result of these activities are scientific opinions for risk managers, developed jointly by independent experts and EFSA staff [13].

Data on acute and chronic toxicity, genotoxicity, carcinogenicity, and allergenicity of various food additives are also analyzed. The collected data and conclusions of the expert panels are regularly reviewed and published in the form of reports (Scientific Opinion on Re-evaluation) [13]. 

Table 1 shows the data on allergenicity, sensitization, hypersensitivity and ADI of antioxidant food colors discussed in the manuscript, based on the scientific reports of the EFSA Experts Panels.

## 5. Summary, Conclusions, and Future Perspectives

Various studies of consumer preferences show that food with intense colors is considered to be healthier, fresher, and qualitatively better. Intensely colored food is much more likely to be chosen by consumers. At the same time, a modern consumer is aware of the risks associated with the use of various additives that improve the appearance, including the color of food, including allergies and anaphylaxis. In addition, a conscious consumer often expects that food can perform not only a nutritional function, but also a pro-health function. In connection with these trends, the multifunctional role of food additives, including dyes, which can also perform various health-promoting functions, is increasingly noticed.

Commonly used food coloring additives, in addition to their coloring function, also have antioxidant properties. Antioxidants are compounds with anti-inflammatory and health-promoting properties. Antioxidants are bioactive compounds found abundantly in various food groups such as vegetables, fruits, nuts, grains, and beverages. Also, extracts from various colored fruits or vegetables are used as food colorings of natural origin. Chronic inflammation plays a key role in the etiology and progression of chronic diseases, including allergies and asthma. A diet rich in antioxidants and other bioactive compounds may modulate the course of inflammation and be an element of pro-health prevention.

Although some food colors, due to their antioxidant properties, seem to be excellent functional food additives, it should be taken into account that they may be a cause of sensitization in predisposed individuals. It seems, however, that although dyes are common food additives, allergy to them is rather rare. It is estimated that sensitization to food dyes in the general population is 0.01–0.23% and is more common in people with atopy (2–7%) [130,131]. Documented allergic reactions to dyes are rather mild and mainly affect the skin and gastrointestinal tract, with anaphylaxis rarely reported. Among the hypersensitivity to food dyes of natural origin, the best documented seem to be allergies to dyes of animal origin, extracted from cactus bugs (*Dactylopius coccus*)-cochineal [132]. Interestingly, according to consumers, natural pigments are rather safe, while synthetic dyes are considered rather harmful [130,131].

The analysis of the available literature data does not indicate that dyes with antioxidant properties, such as carotenoids, anthocyanins, chlorophyll, betanin, and curcumin, cause a significant risk of allergic reactions. On the contrary, as strong antioxidants, they can have a beneficial effect on the body and play an important role in the prevention and treatment of allergies and asthma. This is due to their anti-inflammatory, antioxidant, and immunomodulatory properties, which, although used in medicine since ancient times, are still the subject of intensive research.

It should also be remembered that these dyes are obtained from natural plant sources. This means that they may be contaminated with various proteins from the plants from which these dyes have been extracted. These proteins can be strong allergens and they can be the real cause of allergic reactions after eating foods colored with these dyes. This is an independent cause of sensitization that is unrelated to the dye and its antioxidant properties.

## Figures and Tables

**Table 1:** Data on allergenicity, sensitization, hypersensitivity, and the ADI of food dyes with antioxidant properties that were discussed in the manuscript.

Food Dye (E Number)	Allergenicity/Hypersensitivity/Intolerance	References Indicated in the EFSA Report, Which Were the Basis for the Development of the Scientific Opinion of the EFSA Expert Panel	ADI	Cited EFSA Report [ref. nr]
Anthocyanins (E163)	The EFSA Expert Panel did not consider this issue relevant to the safety of these food additives for humans.	No references indicated.	The ADI for anthocyanins has been established at 0–2.5 mg/kg bw/day. * * *This value was determined for anthocyanins from grape peel.*	[117]
Betanin-beetroot red (E162)	According to the EFSA Expert Panel, the widespread consumption of beetroot red, both in natural food products and as a color additive, and the lack of reports of allergic reactions and intolerances, suggests that betanin is not a significant cause of sensitization, allergy, hypersensitivity, and food intolerance.	A case report of a woman who developed anaphylactic shock during beeturia, but according to the authors, the anaphylaxis was not caused by hypersensitivity to beetroot extract [118].	There is not enough database to establish the ADI. Beetroot red is a natural component of the diet and exposure to betanin ingested as a food additive is not considered to increase the risk associated with the consumption of naturally occurring plant beetroot red.	[119]
		This in vitro study showed reduced production of IgE by rat splenic lymphocytes exposed to betanin of unknown specification at concentrations of 1 μM and 10 μM. Based on these results, the authors concluded that these results suggest that natural dyes have a regulatory effect on immunoglobulin production [120].		
Chlorophylls (E 140(i))	The EFSA notes that sources of chlorophyll (e.g., alfalfa) may contain proteins with homology to some peanut proteins, which are strong allergens. This means that chlorophyll extracts from plants may also have allergenic potential [121].	No reported cases of allergy to chlorophylls (E 140(i)) were found.	Chlorophylls are natural dietary components that occur naturally in many foods in relatively high concentrations. For this reason, exposure from the use of chlorophyll as food additives is lower than exposure to chlorophyll from natural sources. Therefore, it is not necessary to set an ADI.	[122]
Chlorophyllins (E 140(ii))	According to the EFSA, no documented cases of allergy or hypersensitivity to chlorophyllins derived from food have been published until the date of the cited scientific report [123]	No cases of allergy to chlorophyllins (E 140(ii)) have been identified in the literature.	According to the EFSA, due to the lack of adequate data on absorption, distribution, metabolism, excretion, and toxicity, and the fact that chlorophyllins (E 140(ii)) are neither natural components of the normal diet nor metabolites of chlorophylls in humans, the assessment of the safety of chlorophyllins as a food additive is not currently possible, but definitely necessary.	[123]
copper complexes of chlorophylls–Cu-Chlorophylls (E 141(i)) and copper complexes of chlorophyllins; Cu-Chloropchyllins (E 141(ii))	According to the EFSA, the available data do not indicate significant immunotoxicity or allergenic potential of Cu-chlorophylls and Cu-chlorophyllins when used as food additives.	A case of an allergic reaction (recurrent angioedema, rhinoconjunctivitis, and asthma symptoms) was reported in a 28-year-old woman after ingestion of foods containing Cu-chlorophylls (E 141(i)). Hypersensitivity to E141(i) was confirmed by challenge tests using food consumed by the woman and E141(i) dye. It was established that the trigger for swelling was green candy. To trigger the reaction, it was necessary to consume 1 mg of E141(i) or five candies. The elimination of the E141(i) dye from the patient’s diet prevented further episodes of hypersensitivity symptoms [60].	Provisionally established ADI for Cu-Chlorophyllin at 0–15 mg/kg bw/day. * * *According to the EFSA Expert Panel, the current ADI data for copper complexes of chlorophylls and chlorophyllins should be considered provisional and should be reviewed again. This is due to the lack of reliable data on absorption, distribution, metabolism, excretion, and toxicity.*	[124]
mixed carotenes (E 160a (i)) and beta-carotene (E 160a (ii))	According to the EFSA, the available data on hypersensitivity, sensitization, and/or allergy to carotenoids is very limited. For this reason, they do not allow for unambiguous opinions in this regard.	In estimating the allergenic potential of carotenoids, the EFSA refers to analyses of the status of safety assessments of food additives currently authorized in the EU “Food Additives in Europe 2000” (TemaNord 2002). The TemaNord (2002) report describes a research project involving 135 patients with urticaria or atopic dermatitis and 123 patients with contact dermatitis. All patients were orally administered β-carotene (100 mg) together with β-apo-carotene (100 mg). In the first group, one patient developed symptoms of hypersensitivity and one patient had ambiguous symptoms suggesting hypersensitivity. No hypersensitivity response was observed in the second group [125].	The ADI for carotenoids has been established at 0–5 mg/kg bw/day. * * *According to the current EFSA opinion, this value should be re-examined. It should also take into account the share of naturally occurring carotenes in food.*	[126]
Curcumin (E 100)	According to the EFSA Panel, both pro- and antiallergic effects of curcumin have been documented in the available literature reports.	The EFSA notes that several cases of hypersensitivity to curcumin have been reported in the form of contact dermatitis [127] or contact urticaria [128] after the use of cosmetics containing this dye. On the other hand, the EFSA also indicates studies that have demonstrated the immunomodulatory, including antiallergic, effect of curcumin. According to available reports, curcumin has an inhibitory effect on the release of histamine from mast cells. The results obtained in a mouse model of allergy indicate a marked inhibition of the allergic reaction in animals treated with curcumin, suggesting a major role of curcumin in reducing the allergic response [101].	The EFSA Expert Panel has set an ADI for curcumin of 0–3 mg/kg bw/day. * * *The Panel also noted that, in everyday life, curcumin intake from a normal diet is typically less than 7% of the ADI. In some European countries, however, it happens that at maximum consumption levels the ADI is exceeded.*	[129]

Legend: E number—the code of the food additive recognized by the specialized institutions of the European Union as safe and approved for use (the so-called E list); the list of E numbers is compiled by the EFSA and published in Regulation (EC) N° 1333/2008 of the European Parliament and the Council of 16 December 2008 on food additives (in the list of references No. 13). EFSA—European Food Safety Authority. ADI—Acceptable Daily Intake.bw—body weight.

## Data Availability

Not applicable.
